# Scraps

**Published:** 1891-09

**Authors:** 


### SCRAPS

PICKED UP BY THE ASSISTANT-EDITOR.

---

**Shakspere's Family as Patients.**—"Master John Hall, Phyfician," who was in practice at Stratford-on-Avon, married Shakspere's daughter, Susanna, in 1607. In Latin he wrote details of the illnesses of many of his private patients, whose names he gives in full in the clinical records which he published. Under the title of *Select Observations on Englifh Bodies of Eminent Perfons in defperate Diseases*, his book was translated by James Cook, of Warwick, author of the *Marrow of Chirurgery*. The notes of many of Dr. Hall's cases are interesting, not only on account of the family association of the patients, but because they are additional instances of the diagnosis and treatment then current. Polypharmacy was in fashion then, almost more than at the present time, although we have not to go very far now to find those who, like Dr. Hall and his contemporaries, seem to think that there exists a drug to be appropriated to each individual symptom. Although Dr. Hall's diagnosis is often open to criticism, his treatment was based on sound principles. Not seldom his success was obtained by the induction of copious vomiting, and nearly always by very free purgation. In these days of elegant pharmacy we are in danger of forgetting the value of this mode of treatment, and we might do well to follow the example of our ancestors somewhat more often and, on their lines, secure quickly very good results by methods which produce much less discomfort to the patient than in those times of crude therapeutics.

In an attack from which Dr. Hall's wife suffered, a less quantity of nauseous medicine was required than was usual. Here is her husband's account of the illness:

"Mrs. *Hall* of *Stratford*, my Wife, being miferably tormented with the Cholick, was cured as followeth. ℞ *Diaphoen. Diacatholic.* ana ℥i. *Pul. Holand* ʒii. *Ol. Rutæ* ℥i. *Lact. q. f. f. Clyft.* This injected gave her two Stools, yet the Pain continued, being but little mitigated; therefore I appointed to inject a Pint of Sack made hot. This prefently brought forth a great deal of Wind, and freed her from all Pain. To her Stomach was applied a Plaifter *de Labd. Crat. cum Caran. & Spec. Aromat. rofat. & Ol. Macis.* With one of thefe Clyfters I delivered the Earle of *Northampton* from a grievous Cholick."

The virtues of sack have been celebrated by Jack Falstaff in a well-known passage, although he would no doubt have considered that to use it by this route was a deliberate waste of his favourite liquor. But it seems to have done more good when directed by the hands of Dr. Hall than it ever did in the mouth of Jack.

The health of Dr. Hall's daughter, when she was sixteen years of age, was a source of anxiety to her parents. Her father thus records the difficulties:

"*Elizabeth Hall*, my only Daughter, was vexed with *Tortura Oris*, or the Convulfion of the Mouth, and was happily cured as followeth: Firft, I exhibited thefe Pills: ℞ *Pil. Coch. & Aurear.* ana ʒi. *f. Pil.* 10. She took five the firft day, which gave her feven Stools; the next day with the other five fhe had five ftools. I fomented the part with *Theriac. Andromac.* and *Aq. Vitæ.* To the Neck was ufed this: ℞ *Unguent. Martiat. magn.* ℥i. *Ol. Laurin. Petrolei, Caftor. & Terebinth.* ana ʒfs. *de lateribus* ʒfs. *Mifc.* By this fhe had great advantage, her Courfes being obftructed. Thus I purged her: ℞ *Pil. fœtid.* ʒi. *Caftor.* ʒi. *de Succin. Rhab. agaric.* ana ℈ifs. *f. Maff.* She took of this five Pills in the morning, of the bignefs of Peafe; they gave eight ftools. The next day fhe took *Aq. Ophthalm.* fee *Obfer.* 3, as ℞ *Tutiæ.*, &c., her Courfes flowed. For an *Ophthalmia*, of which fhe laboured, I ufed our Ophthalmick Water, dropping two or three drops into her Eye. Her Courfes ftaying again, I gave the following Sudorific Decoct. ℞ *Lign. Vitæ* ℥ii. *Saffafras* ℥fs. *Saffap.* ℥i. *Chin.* ʒvi. *macerat. per* 24 *hor. in Aq. fontan.* lb. viii. *After boyl them to* lb. iv. After the ufe of thefe, the former form of her Mouth and Face was reftored (there was not omitted *Ol. Sarfap.*, which was above all to anoint the Neck.) *Jan.* 5, 1624.

"In the beginning of *April* fhe went to *London*, and returning homewards, the 22d of the faid Month, fhe took cold, and fell into the faid Diftemper on the contrary fide of the Face; before it was on the left fide, now on the right; and although fhe was grievoufly afflicted with it, yet by the bleffing of God fhe was cured in fixteen days, as followeth: ℞ *Pil. de Succin* ʒfs. *Aurear.* ℈i. *f. Pil.* v. She took them when fhe went to bed. The fame night her Neck was anointed with Oil of *Saffafr.* In the morning I gave ʒfs. of *Pil. Ruffi.*, and again ufed the faid Oil with *Aqua Vitæ*, and dropped into her Eye the Ophthalmick Water. The aforefaid Oil being wanting, I ufed the following: ℞ *Pul. Caftor. Myrrh. Nuc. Mofch. Croci.* ā ℈i. *Ol. Rutæ, Laurin. Petrol. Tereb.* ā ʒii. *Ungu. martiat.* ℥fs. *Ol. Coftin. de Peper.* ā ʒi. *Mifc.* But firft the Neck was fomented with *Aqua Vitæ*, in which was infufed *Nutmegs, Cinnamon, Cloves, Pepper.* She eat Nutmegs often. To the Noftrils, and top of the Head was ufed the Oil of Amber. She chewed on the found fide, Pellitory of *Spain*, and was often purged with the following Pills: ℞ *Pill. fœtid.* ℈i. *Caftor pul.* ℈fs. *Pil. Ruffi. & de Succin.* ā ℈i. *f. Pil.* N° v. And thus fhe was reftored.

"In the fame year, *May* 24. fhe was afflicted with an Erratick Feaver; fometimes fhe was hot, and by and by sweating, again cold, all in the fpace of half an hour, and thus fhe was vexed oft in a day. Thus I purged her: ℞ *the Roots of Parfly*,

*Fennel, each* Mſs. *Elder Bark* Mii. *Roots of the vulgar Oris, of Madder,* each Mi. *Roots of Sparagus,* Mii. *Boyl them in ſufficient quantity of Water to ſix pints. To the ſtraining, add Rubarb, Agarick, each* ℥ſs. *Sena* ℥vi. *Mechoacan* ℥ii., *Calamus Aromaticus* ℥i. *Aniſeeds* ℥i., *Cinnamon* ℥ſs. *Infuſe them in a Veſſel well ſtopt according to art: ſtrain it again, and to the ſtraining add Sugar ſufficient to make a Syrup. of this take* ℥iv. *Rubarb infuſed in* ℥v. *of Cichory water* ʒii. *Mix them, and give ſeven ſpoonfuls every day faſting.* It gave ſeven or eight ſtools without pain. ℞ *Sarſap.* ℥i., *Saſſafr.* ʒii., *Guaiac* ℥i., *Liquoris* ℥ſs., *Herb of Succory, Sage, Roſemary, each* Mſs. *Boyl them in ten pints of Water till half be waſted.* Of which ſhe took a draught hot in the morning. The following was uſed to anoint the Spine: ℞ *Gum. Galban. Bdel. diſſol. in Aq. Vit.* ā ℥ſs. *Benzoin.* ℥i. *Styrac. liquid.* ʒi. *Fol. Rut. Chamœpith. Flor. Stœchad. Lavendula,* ā ʒii. *Rad. coſti.* ℥ſs., *Caſtorei* ℈i. *infund. miſc. & pulveriſat. in Aq. Vitæ.* It is to be infuſed in ſome hot place for ſome days. Before it was uſed, the Spine was rubb'd. An hour after it was uſed, all the Symptoms remitted daily till ſhe was well. Thus was ſhe delivered from Death, and deadly Diſeaſes, and was well for many years. To God be praiſe."

The *Aq. Ophthalm.*, concerning which Dr. Hall's reader was referred to Observation III., was made thus:—"℞ *Sarcocol* waſh'd, ʒiij. *Prepared Tutty* ʒij. *Aloes* ʒj. *White Sugar-candy* ʒiſs. *Saffron* gr. iv. *Roſewater* ℥iv. Mix them, letting them ſtand a day, ſhaking them oft."

Dr. Hall did not shrink from administering drugs to himself as freely as he gave them to his patients. When fifty-six years of age (he was only twelve years younger than his father-in-law) he had "an immoderate Flux of the Hemorrhoids," the sequelæ of which nearly killed him. His remedies had, however, done him much good before the arrival of the two physicians for whom his wife sent.

The identification of much of the Materia Medica in Dr. Hall's prescriptions is difficult, and any information about the less known things would be welcome.

The abbreviation M. was used to signify a handful.

Amongst Dr. Hall's other patients was Mrs. Nash, probably the mother of his daughter's first husband. She had "of a long time laboured of a Conſumption, and now afflicted with Wind of the Stomach, as alſo Heat thereof, with ſweating from the Pit of the Stomach to the Crown of the Head, having great Pain of the Head, eſpecially after Meat." He used remedies which "freed her from Wind, and ſhe was able to eat, and ſaid ſhe was very well for a long time after."

It is also likely that the husband of Shakspere's other daughter, Judith, came under Dr. Hall's care, for he attended "Mr. *Queeny*, labouring of a grievous Cough, with vomiting abundance of Phlegm and Meat, having a gentle Feaver, being very weak, and had red Urine without ſediment." His malady was not amenable to the evacuant treatment which was generally so successful in Dr. Hall's hands; and although the illness somewhat abated, "being not wholly freed from it, he fell into it again the next year, and all Remedies proving ſucceſsleſs, he died. He was a Man of a good Wit, expert in Tongues, and very learned."

It is of further interest to note that another of Dr. Hall's patients was the author of the *Poly-Olbion*, whose monument in Westminster Abbey shows by its inscription how highly he was esteemed by his contemporaries, but whose fame was already dim in the days of Goldsmith, who, upon seeing his grave in the Abbey, exclaimed, "Drayton! I never heard of him before." Upon one occasion Dr. Hall quickly brought him round from an illness which is thus described: "Mr. *Drayton*, an excellent Poet, labouring of a Tertian, was cured by the following: ℞ *the Emetick Infuſion* ℥i. *Syrup of Violets a ſpoonful: mix them.* This given, wrought very well both upwards and downwards."

